# Melatonin Ameliorates Apoptosis of A549 Cells Exposed to Chicken House PM_2.5_: A Novel Insight in Poultry Production

**DOI:** 10.3390/toxics11070562

**Published:** 2023-06-28

**Authors:** Pengyuan Dai, Jiakun Shen, Dan Shen, Xiaotong Li, Tin-Tin Win-Shwe, Chunmei Li

**Affiliations:** 1 Institute of Reproductive Medicine, Medical School of Nantong University, Nantong 226019, China; pengyuandai@ntu.edu.cn; 2 Research Center for Livestock Environmental Control and Smart Production, College of Animal Science and Technology, Nanjing Agricultural University, 1 Weigang, Nanjing 210095, China; shenjiakun2008@126.com (J.S.); 2018205016@niau.edu.cn (D.S.); 2019105052@njau.edu.cn (X.L.); 3 Center for Environmental Risk Research, National Institute for Environmental Studies, Tsukuba 305-8506, Japan; tin.tin.win.shwe@nies.go.jp

**Keywords:** melatonin, particulate matter 2.5, broiler breeding house, cell survival, endoplasmic reticulum stress, oxidative stress

## Abstract

The particulate matter 2.5 (PM_2.5_) from the chicken production system can cause lung injury and reduce productivity through prolonged breath as it attaches large amounts of harmful substances and microbes. Melatonin has acted to regulate physiological and metabolic disorders and improve growth performance during poultry production. This research would investigate the apoptosis caused by chicken house PM_2.5_ on lung pulmonary epithelial cells and the protective action of melatonin. Here, the basal epithelial cells of human lung adenocarcinoma (A549 cells) were subjected to PM_2.5_ from the broiler breeding house to investigate the apoptosis induced by PM_2.5_ as well as the alleviation of melatonin. The apoptosis was aggravated by PM_2.5_ (12.5 and 25 μg/mL) substantially, and the expression of *Bcl-2*, *Bad*, *Bax*, *PERK*, and *CHOP* increased dramatically after PM_2.5_ treatment. Additionally, the up-regulation of cleaved caspase-9 and cleaved caspase-3 as well as endoplasmic reticulum stress (ERS)-related proteins, including ATF6 and CHOP, was observed due to PM_2.5_ exposure. It is worth noting that melatonin could support A549 cells’ survival, in which reduced expression of *Bax*, *Bad*, cleaved caspase-3, and cleaved caspase-9 appeared. Concurrently, the level of malondialdehyde (MDA) was down-regulated and enhanced the intracellular content of total superoxide dismutase (T-SOD) and catalase (CAT) after treatment by PM_2.5_ together with melatonin. Collectively, our study underlined that melatonin exerted an anti-apoptotic action on A549 cells by strengthening their antioxidant capacity.

## 1. Introduction

Melatonin, as an indoleamine naturally occurring, mainly functions as a regulator in the sleep/wake cycle and other circadian and seasonal rhythms [1], and plays a part in photoperiodic response, immunity enhancement, the anti-aging process, and anti-oxidation [2,3,4,5]. Studies in terms of melatonin in poultry production have been conducted extensively. It has been used as a key mediator in poultry reproductive actions [6,7], increasing the laying quantity of layers [8], egg weights [9], bone strengthening [10], and improving the daily gain of broilers [11]. Further, it should be noted that melatonin, serving as an antioxidant, can inhibit oxidative stress effectively not only by scavenging reactive oxygen species (ROS) directly but also by upregulating antioxidant enzyme activity [12,13,14].

During intensive poultry production, a large scale of particulate matter (PM) was generated, the sources of which included feed, feces, animal dander, skin feathers, urine, and padding [15,16]. The intensive feeding posed a heavier burden on animals and workers and also contributed to atmospheric pollution through exhaust emissions [17]. It has been reported that there was a high incidence of respiratory diseases in intensive poultry houses, further resulting in poor immune performance and body weight [18,19], with increased mortality and economic losses observed [17,20]. Our latest research highlighted the exposure of chickens to PM from broiler house-induced lung inflammation, with visible changes in microbial and metabolic disorders associated with lung damage [21]. Additionally, the existing evidence revealed that PM exposure is strongly correlated with lung diseases [22,23]; however, effective measures to relive such stress are still lacking.

Particulate matter 2.5 (with an aerodynamic diameter of ≤2.5 µm, PM_2.5_) has a larger surface area and carries various toxic substances. Epidemiological investigations have shown that PM_2.5_ increases susceptibility to pulmonary infection, chronic obstructive pulmonary disease, and bronchial asthma [24,25]. Our earlier research indicated the concentration of PM_2.5_ from chicken houses exceeded the annual average concentration of PM_2.5_ from the atmosphere, and the composition of PM_2.5_ in the chicken houses included organic carbons, ions, and endotoxin, as well as a large number of harmful bacteria and fungi [16], which is different from that in the atmosphere [26]; therefore, the discrepant biological consequences imposed by PM_2.5_ were indicated. Besides, the issue of whether melatonin relieves lung damage inflicted by PM_2.5_ in animal production remains elusive. Here, we focused on the cell damage induced by chicken house PM_2.5_ in A549 cells and further investigated the alleviating effect of melatonin on apoptosis in A549 cells. This project aimed to explore the detrimental effect of PM_2.5_ from poultry housing on the respiratory system in vitro, but more than that, it offers evidence of remission of the lung injury exerted by melatonin. We look forward to providing a theoretical basis for improving poultry production.

## 2. Materials and Methods

### 2.1. PM_2.5_ Sampling and Extraction

The PM_2.5_ in the chicken house was collected on a filter membrane (Whatman, Piscataway, NJ, USA) by a BTPM-HS1 atmosphere particle sampler (Dandong Baxter Co., Ltd., Dandong, China). The PM_2.5_ on filter membranes was oscillated into deionized water and then filtered with six layers of gauze before centrifugation. After drying, the PM_2.5_ was dissolved into saline and then sterilized before being stocked at −20 °C.

### 2.2. Cell Culture and Treatment

The A549 cell line (ATCC, Manassas, VA, USA) was used for exposure assays. The A549 cells were cultured in an F12 medium (Gibco, Waltham, MA, USA) supplemented with 10% fetal serum (BI, Kibbutz Beit-Haemek, Israel) and 0.5% penicillin-streptomycin-amphotericin B solution (Sigma, New York, NY, USA). Cells were kept at 37 °C with 5% CO_2_. The humidity of the incubator was maintained by the water tray at the bottom. The PM_2.5_ (1 mg/mL) and the stocked melatonin (10 mmol/mL) (MedchemExpress, Monmouth Junction, NJ, USA) were diluted by the medium at different concentrations (6.25 μg/mL, 12.5 μg/mL, 25 μg/mL for PM_2.5_, and 0, 12.5 μmol, 25 μmol, 50 μmol, 100 μmol, 200 μmol, 400 μmol, 800 μmol, 1600 μmol, and 3200 μmol for melatonin) and the cells were treated after the confluence reached 80%. In the cell cycle, cell apoptosis, transmission electron microscope (TEM), RT-PCR, and Western blot assays, the cells were cultured into a 6-cell plate, and then exposed to PM_2.5_ or melatonin; as for the methyl thiazolyl tetrazolium (MTT) assay, the cells were detached into a 96-cell plate and then treated by PM_2.5_ or melatonin.

### 2.3. The Detection of the Cell Cycle, Cell Apoptosis, and Transmission Electron Microscopy (TEM)

For cell cycle detection, A549 cells were washed with ice-cold PBS three times after being digested by trypsin (Gibco, Waltham, MA, USA). Then, the cells were fixed with 70% ethanol for 8 h and stained with propidium iodide (PI) (Vazyme Biotech Co., Ltd., Nanjing, China) for 10 min in the dark. In cell apoptosis, cells were detached with trypsin-excluded EDTA (Gibco, Waltham, MA, USA) and washed with ice-cold PBS three times. Cells were centrifuged at 300× *g* at 4 °C for 5 min and then stained with 5 μL of Annexin V-fluorescein isothiocyanate (FITC) and 5 μL of PI (Vazyme Biotech, Nanjing, China, A211-01/02) for 10 min in the dark. Each sample was added to 400 μL of 1 × binding buffer before detection. Cell cycle and cell apoptosis were determined by flow cytometry (BD, Franklin Lakes, NJ, USA).

The cells were collected and then washed with PBS three times. A549 cells were fixed with 4% glutaraldehyde at 4 °C for 72 h, then rinsed again with PBS and refixed with 1% osmium tetroxide for 30 min at room temperature. The following steps proceed based on the procedure described by Winey et al. [27].

### 2.4. Real-Time PCR and Western Blot Assays

A549 cells were lysed with TRIzol^®^ reagent (Takara, Ichikawa, Japan). RNA was extracted by chloroform (Nanjing Jiancheng Biological Engineering Institute, Nanjing, China) and isopropanol (Jiancheng), and the RNA was washed with 75% ethanol (Jiancheng) two times. For the Western blot, cells were lysed with cell lysis buffer containing 1% phenylmethanesulfonyl fluoride (PMSF) (Solarbio, Co., Ltd., Beijing, China). The protein concentration was detected by the BCA protein concentration detection commercial kit (Sigma). The purity and concentration of RNA were detected by NanoDrop^®^ ND-1000 (Thermo, Waltham, MA, USA). RNA integrity was detected by 0.4% agarose gel electrophoresis. The reverse transcription was performed by the SuperScript (SS) First-Strand Synthesis System (Takara, Berkeley, CA, USA). Real-time PCR (RT-PCR) was conducted on the Applied Biosystem StepOneM Real-Time PCR system (ABI, Los Angeles, CA, USA) using SYBR Green Master Mix (Takara, Berkeley, CA, USA). The RT-PCR procedure was as follows: 95 °C for 30 s, 95 °C for 5 s and 34 s at 60 °C for 40 cycles, and then 95 °C for 15 s, 60 °C for 1 min, and 95 °C for 15 s. The product’s specificity was determined by melting curve analysis. Every gene in each sample was detected three times. The data processing was performed by the ΔΔCt method. The gene primers are shown in Table 1.

The protein was degraded by boiling for 5 min after mixing with loading buffer (Beyotime Biotech, Co., Ltd., Shanghai, China). 12% agarose gels were used for protein electrophoresis at 140 V for 55 min, and then the transferring process was performed at 90 v for 70 min. The protein was blocked by 5% bovine serum albumin for 2 h, then incubated with antibodies overnight at 4 °C. The primary antibodies included glucose-regulating protein (GRP78) (CST, Danvers, MA, USA), C/EBP-homologous protein (CHOP) (CST), activating transcription factor 6 (ATF6) (CST), cleaved caspase-3 (CST), cleaved caspase-9 (CST), and NF-E2-related factor 2 (Nrf2) (CST). The second antibody includes HRP-conjugated anti-rabbit or mouse IgG (Biosharp, Suzhou, China). β-actin (CST) was used as the internal control.

### 2.5. The Detection of Cell Viability

The A549 cells were cultured in the 96-cell plate for 24 h and then treated with melatonin at different concentrations (0, 12.5 μmol, 25 μmol, 50 μmol, 100 μmol, 200 μmol, 400 μmol, 800 μmol, 1600 μmol, and 3200 μmol). After 12 h, the supernatant was discarded, and 100 μL of MTT (Jiancheng) was added for another 4 h. Then, after discarding MTT, the cells were incubated with 150 μL of dimethyl sulfoxide (DMSO) per well for 15 min at room temperature. The absorbance was detected by a microplate reader (Thermo Scientific, Waltham, MA, USA).

### 2.6. The Detection of T-SOD, CAT, and MDA

The activity of total superoxide dismutase (T-SOD, A001-1-2), catalase (CAT, A007-1-1), and malondialdehyde (MDA, A003-1-2) was detected by the xanthine oxidase, ammonium molybdate colorimetric, and thiobarbituric acid methods, respectively, using the commercial kit (Jiancheng) and based on the manufacturer guidelines.

### 2.7. Statistical Analysis

The data were analyzed by GraphPad Prism 7.0 (GraphPad Software, San Diego, CA, USA), and the data were shown as means ± standard deviation (SD) depending on at least three independent experiments. The difference between the two groups was determined by a t-test. One-way ANOVA performed with the Tukey test was adopted in comparisons of more than two groups, either between the control group and the treated group or between any two-column means. *p* < 0.05 represents a significant difference between the two groups.

## 3. Results

### 3.1. The Effects of PM_2.5_ on the Cell Cycle Distribution and Cell Apoptosis of A549 Cells

**Figure 1:**
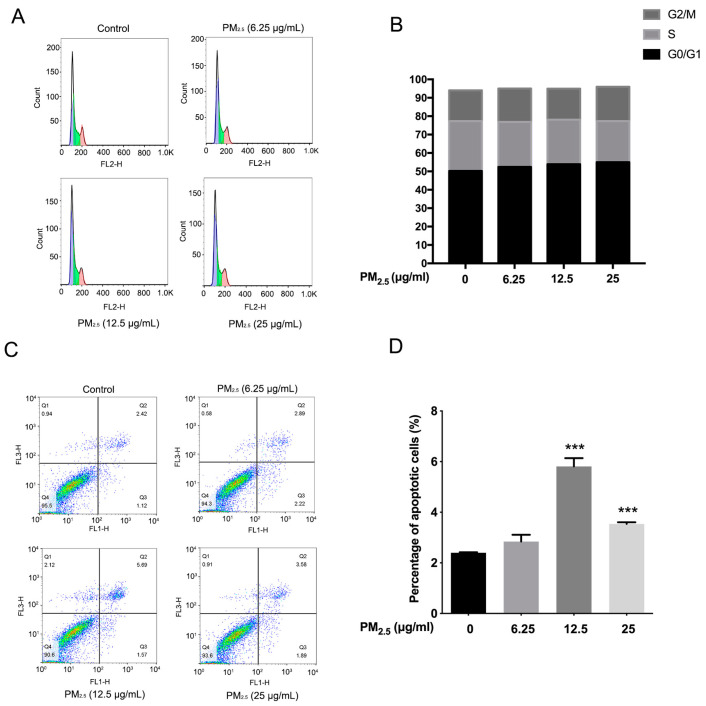
The effect of PM_2.5_ on the distribution of cell cycle and apoptosis in A549 cells. (**A**) The cell cycle was determined by flow cytometry after PM_2.5_ treatment at concentrations of 6.25 μg/mL, 12.5 μg/mL, and 25 μg/mL for 12 h. (**B**) Quantification of cell cycle distribution after PM_2.5_ treatment, n = 3. (**C**) Apoptosis was detected by flow cytometry after exposure to PM_2.5_ at different concentrations (0, 6.25 μg/mL, 12.5 μg/mL, 25 μg/mL) for 12 h, n = 3. (**D**) Quantification of the percentage of apoptotic cells after PM_2.5_ treatment. *** *p* < 0.001, n = 3.

The chicken house for PM_2.5_ sampling, the season, the chicken ages in-house, and the PM_2.5_ extraction methods were consistent with our previous studies [16,28]. The primary components of PM_2.5_ included organic carbon, elemental carbon, NO_3_^−^, NH_4_^+^, SO_4_^2−^, Cl^−^, Ca^2+^, K^+^, Na^+^, F^−^, and endotoxin (Table 2) [16]. In cell cycle and cell apoptosis, A549 cells were treated with PM_2.5_ at concentrations of 6.25 μg/mL, 12.5 μg/mL, and 25 μg/mL, for 12 h. The treatment concentration and time of PM_2.5_ were based on our previous study [28]. The results show that the proportion of G0/G1 phase cells gradually increased while the S phase cells decreased after treatment with PM_2.5_. A549 cells in the G0/G1 phase were 50.2% ± 5.3, 52.4% ± 3.8, 53.9% ± 3.2, and 55.0% ± 2.8, and cells in the S phase were 27.1% ± 4.9, 24.4% ± 2.6, 24.1% ± 3.2, and 22.3% ± 4.0 at 6.25 μg/mL, 12.5 μg/mL, and 25 μg/mL in the treated groups, respectively (Figure 1A,B). As shown in Figure 1C,D, the apoptotic cells increased significantly after exposure to PM_2.5_ at 12.5, and 25 μg/mL (Figure 1D). The results indicate that PM_2.5_ from chicken houses induces apoptosis in A549 cells.

**Figure 2:**
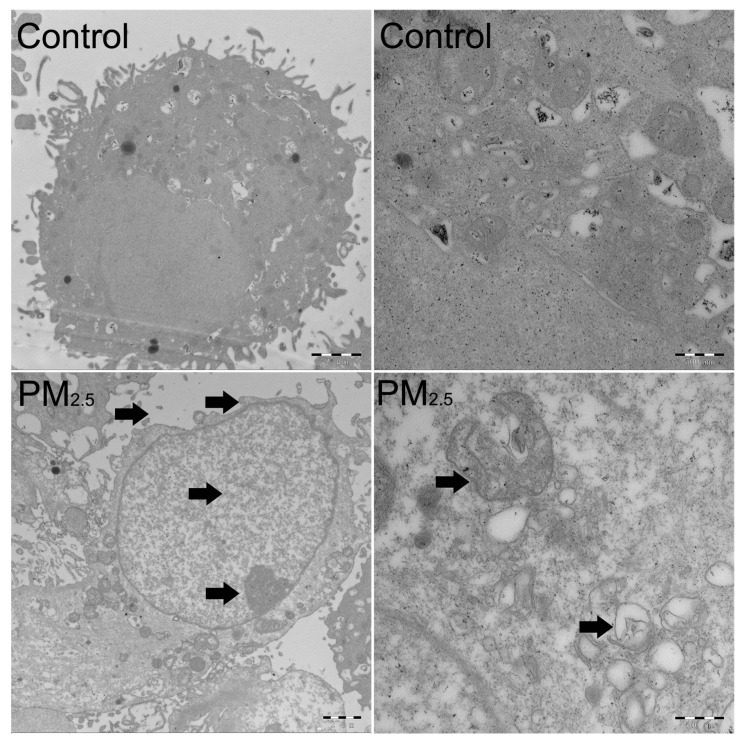
The ultrastructure observation of A549 cells exposed to PM_2.5_. The subcellular structure of A549 cells exposed to PM_2.5_ (25 μg/mL) for 12 h by transmission electron microscope (TEM). Black arrows indicate the apoptosis characteristics of the A549 cell. The arrows indicated nuclear enlargement, chromatin condensation, cytoplasm shrinkage, and cell surface microvilli fracture in the bottom left panel, and organelles swelling into vacuoles in bottom right panel. The scale bars are 2 μm and 500 nm with magnifications of 5000× and 20,000× in the left and right panels, respectively.

**Figure 3:**
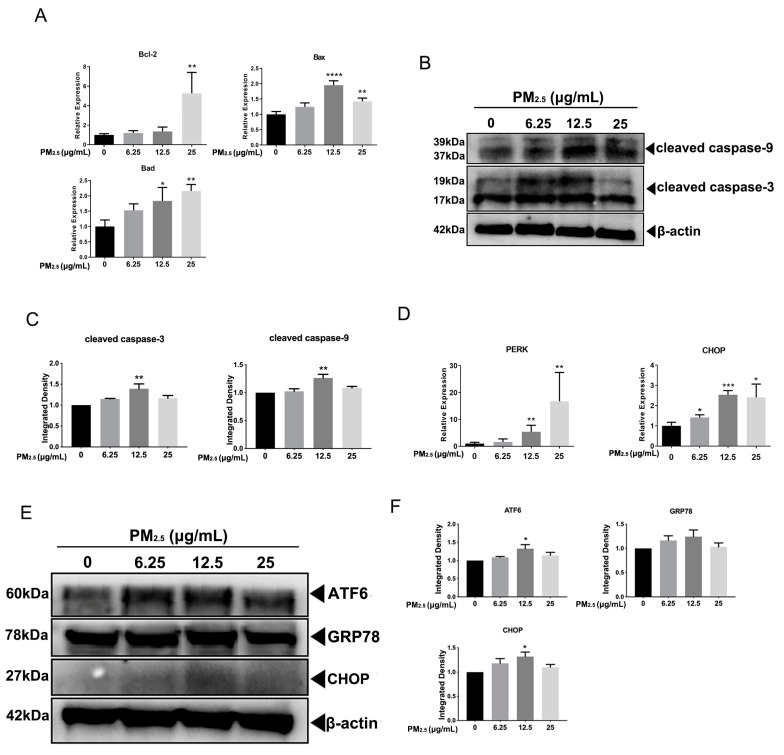
PM_2.5_-induced apoptosis and endoplasmic reticulum stress in A549 cells. (**A**) The analysis of genes related to apoptosis, including *Bcl-2*, *Bax*, and *Bad*, in A549 cells exposed to PM_2.5_ at 6.25 μg/mL, 12.5 μg/mL, and 25 μg/mL for 12 h. (**B**) The immunoblotting analysis detected the levels of cleaved caspase-3 and cleaved caspase-9 in A549 cells with the same treatment as shown above. The data shown are representative of three independent experiments. (**C**) The quantitative analysis of cleaved caspase-3 and cleaved caspase-9 at the protein level. (**D**) The expression of *PERK* and *CHOP* was detected in A549 cells exposed to PM_2.5_ at 6.25 μg/mL, 12.5 μg/mL, and 25 μg/mL for 12 h. (**E**) The levels of ATF6, GRP78, and CHOP were determined by immunoblotting analysis in A549 cells with the same treatment as shown above. The data shown are representative of three independent experiments; (**F**) The quantitative analysis of ATF6, GRP78, and CHOP at the protein level; * *p* < 0.05, ** *p* < 0.01, *** *p* < 0.001, **** *p* < 0.0001, n = 3.

The subcellular structure of A549 cells was observed by transmission electron microscopy (TEM). As shown in Figure 2, PM_2.5_ exposure induces nuclear enlargement, chromatin condensation, cytoplasm shrinkage, and cell surface microvilli fracture (as the arrows in the bottom left panel of Figure 2 indicate, at 5000×), which suggests that cells were going to undergo apoptosis. Notably, many organelles in the cytoplasm were swelled into vacuoles (as the arrows in the bottom right panel of Figure 2 indicate, at 20,000×), and mitochondria and endoplasmic reticulum with regular shapes are not in sight. We speculated that PM_2.5_ not only induces cell apoptosis but also causes endoplasmic reticulum stress (ERS) in A549 cells. Moreover, the expression of *Bcl-2*, *Bax*, and *Bad* increased significantly at 25 μg/mL in the A549 cells (*p* < 0.05) (Figure 3A). Consistently, the levels of cleaved caspase-3 and cleaved caspase-9 increased at 12.5 μg/mL, while showing a declining level at 25 μg/mL after exposure to PM_2.5_ (Figure 3B).

### 3.2. PM_2.5_ Caused ERS in A549 Cells

The protein kinase R-like ER kinase (PERK) increased considerably at 12.5 μg/mL and 25 μg/mL in the PM_2.5_-treated group (*p* < 0.01). The expression of CHOP increased significantly in all exposed groups in the A549 cell (*p* < 0.05) (Figure 3D). There was no change in the expression of GRP78 among all groups. The levels of ATF6 and CHOP were expressed in the same way, which elevated gradually while declining at 25 μg/mL of PM_2.5_ in A549 cells (Figure 3E).

### 3.3. Melatonin Alleviated Apoptosis and Improved Cell Antioxidation after Exposure to PM_2.5_ from Chicken Houses

**Figure 4:**
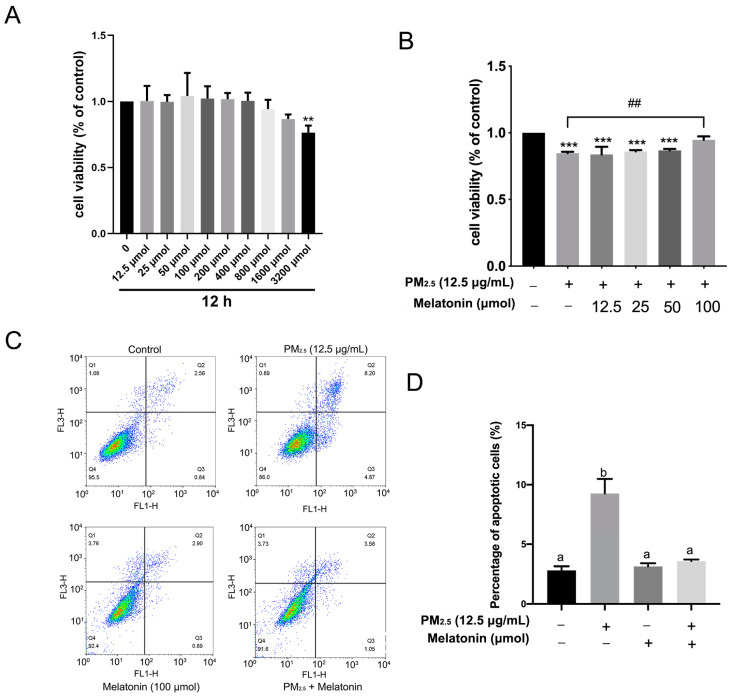
Melatonin resisted apoptosis caused by PM_2.5_ in the A549 cells. (**A**,**B**) indicate the effect of melatonin and the cotreatment of melatonin with PM_2.5_ on A549 cell viability, respectively. ** *p* < 0.01, *** *p* < 0.001, ^##^ *p* < 0.01, n = 4 (A), n = 3 (**B**). The values shown in panels A and B represents the ratio of absorbance value from each group/control. (**C**) The effect of melatonin on apoptosis of A549 cells stimulated by PM_2.5_ via flow cytometry. (**D**) The quantification of the percentage of apoptotic cells after treatment. The different letters in the columns represent significant differences, n = 3.

In this assay, the A549 cells were exposed to melatonin at concentrations of 12.5 μmol, 25 μmol, 50 μmol, 100 μmol, 200 μmol, 400 μmol, 800 μmol, 1600 μmol, and 3200 μmol for 12 h. As shown in Figure 4A, melatonin at 50 μmol and 100 μmol resulted in an increasing tendency on A549 cell viability, and the IC50 is 3.31 × 10^4^ μmol. Further assays show that melatonin at 100 μmol improved the proliferation of A549 cells (*p* < 0.01) (Figure 4B). Consistent with the above results, melatonin at 100 μmol prevented A549 cells from apoptosis significantly after exposure to PM_2.5_, as shown in Figure 4C,D.

**Figure 5:**
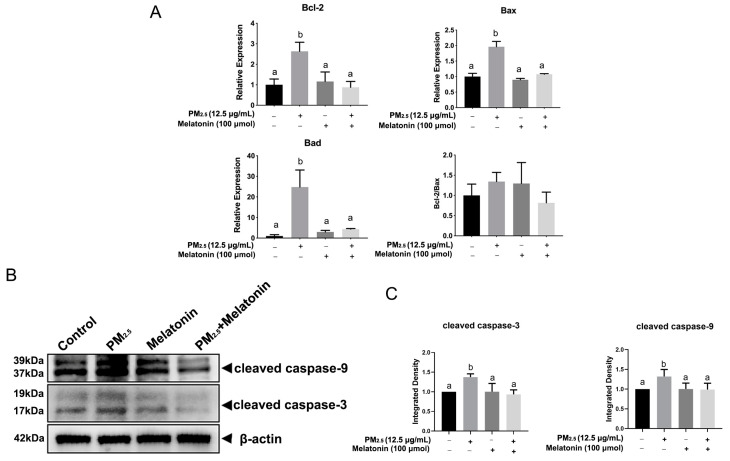
The effect of melatonin on apoptosis of A549 cells. (**A**) The expression of *Bcl-2*, *Bad*, and *Bax* in A549 cells exposed to PM_2.5_ (12.5 μg/mL), melatonin (100 μmol) or co-treatment with PM_2.5_ and melatonin for 12 h. The different letters in the columns indicate significant differences, n = 3. (**B**) The detection of cleaved caspase-9 and cleaved caspase-3 by immunoblotting assay in A549 cells with the same treatment as shown above. The data shown are representative of three independent experiments. The effect of melatonin on the antioxidant capacity of A549 cells treated by PM_2.5_. (**C**) The quantitative analysis of cleaved caspase-3 and cleaved caspase-9 at the protein level. The different letters in the columns indicate significant differences.

**Figure 6:**
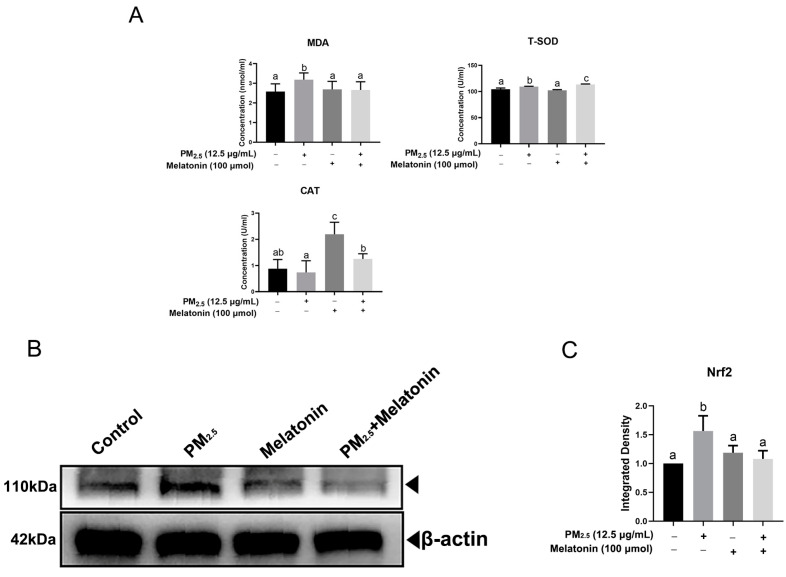
The effect of melatonin on the antioxidation of A549 cells. (**A**) The levels of MDA (n = 5), T-SOD (n = 3), and CAT (n = 7) were detected in supernatant from groups treated by PM_2.5_ (12.5 μg/mL), melatonin (100 μmol), and PM_2.5_ with melatonin. (**B**) The expression of Nrf2 was determined by an immunoblotting assay in A549 cells with the same treatment, as shown above. The data shown are representative of three independent experiments. (**C**) The quantitative analysis of Nrf2 at the protein level. The different letters in the columns indicate significant differences.

Figure 5A shows that melatonin reduced the expression of *Bcl-2*, *Bad*, and *Bax* dramatically in comparison with that in the PM_2.5_-exposed group. There was a lower expression of cleaved caspase-9 and cleaved caspase-3 upon the combined treatment of melatonin with PM_2.5_ compared to that in the PM_2.5_-treated group (Figure 5B). PM_2.5_ stimulation increased T-SOD and CAT levels in A549 cells (Figure 6A). The melatonin stimulation reduced the levels of MDA (*p* < 0.05) and Nrf2 after exposure to PM_2.5_ (Figure 6A,B). Importantly, melatonin improved the release of T-SOD and CAT considerably in PM_2.5_-treated cells (Figure 6A).

## 4. Discussion

The mitochondria play a vital role in regulating apoptosis [29]. Bad and Bid are activated after receiving intracellular death signals and then changing the conformation of *Bax*. *Bax* inserts into the outer membrane of mitochondria and changes the mitochondrial membrane permeability, leading to the release of apoptotic factors such as cytochrome C, which can activate caspase-9 and caspase-3, triggering a series of downstream apoptotic reactions. *Bcl-2*, as an anti-apoptotic gene, acts on the outer membrane of the mitochondria to maintain its integrity [30]. Our earlier research demonstrated that PM_2.5_ from chicken houses contained endotoxin and a large number of organic compounds [16]. Emerging evidence has demonstrated endotoxin-induced apoptosis in many cell lines [31,32,33]. Here, chicken house PM_2.5_ induced A549 cell apoptosis by enhancing the expression of *Bad* and *Bax* and activating cleaved caspase-9 and cleaved caspase-3.

ERS is well characterized by protein misfolding and the accumulation of unfolded proteins, and an internal calcium loss or calcium overload, during which many sensors could be activated, including PERK and ATF6, which are released by GRP78 and aim to recover homeostasis [34]. PERK and ATF6 can activate CHOP, the function of which promotes the expression of *Bad* and *Bax* and suppresses *Bcl-2* and *Bcl-xl*, further inducing apoptosis [35,36]. In this study, chicken house PM_2.5_ caused ERS and promoted the expression of *PERK* and *CHOP*. Furthermore, immunoblotting assays indicate that ATF6 and CHOP levels increased after stimulation by PM_2.5_ in A549 cells. In summary, PM_2.5_ from chicken houses induces ERS and then causes apoptosis through the PERK/ATF6-CHOP-caspase-3 signal pathway. It is worth noting that the expression of ATF6 and CHOP decreased with the concentration of PM_2.5_ at 25 μg/mL in A549 cells, which was consistent with the expression pattern of cleaved caspase-3 and cleaved caspase-9, further demonstrating that their expression is dose-dependent.

The increase of *Bcl-2* and decrease of *Bax* indicate the cells resistance to apoptosis [37]. Studies show melatonin has anti-apoptotic properties by improving *Bcl-2* expression and resisting *Bax* levels. In human monocytic U937 cells irradiated by ultraviolet, melatonin prevented apoptosis by improving the *Bcl-2* level and declining the release of cytochrome C [38]. Melatonin exerts protection against neurodegenerative diseases, including experimental stroke, Parkinson’s disease, and Alzheimer’s disease, by resisting apoptosis by elevating *Bcl-2* and *Bcl-xl* expression and inhibiting *Bax* levels [39,40,41,42]. In this study, melatonin promoted cell proliferation by downregulating the expression of *Bad* and *Bax*, as well as the levels of cleaved caspase-9 and cleaved caspase-3. Notably, the expression of *Bcl-2* also declined in A549 cells by melatonin exposure. We speculated that melatonin stimulation increased other anti-apoptotic members of the *Bcl-2* family, such as *Bcl-xl* and *Mcl-1*. Moreover, Zhou et al. have demonstrated that melatonin in high concentrations can inhibit the viability and migration of A549 cells [43]. Another study indicated that melatonin showed anti-tumor action by resisting the expression of vascular endothelial growth factor (VEGF), which is involved in angiogenesis [44]. Herein, melatonin decreased *Bcl-2* levels, which may prevent the excessive proliferation of A549 cells.

Endotoxin has been demonstrated as an induction agent in some studies [45,46]. Fe from PM_2.5_ is related to DNA breaks in BEAS-2B cells [47] and causes oxidative stress dependent on lipid peroxidation [48]. Here, endotoxin and Fe were detected in PM_2.5_ by component analysis [16], and led to MDA levels increasing significantly after PM_2.5_ treatment, the result of which is similar to our former detection that increased ROS content was observed in A549 cells exposed to PM_2.5_ [28]. Previous studies have well established that oxidative stress can induce signal transduction pathways involved in apoptosis initiated by mitochondria [1,49]. Melatonin, as an antioxidant, has been employed in many assays demonstrating its anti-oxidative capacity [12,50]. Furthermore, the study indicated that melatonin, as a targeting molecule for mitochondria, exerted a protective role on mitochondria by scavenging ROS and inhibiting the mitochondrial permeability transition pore (MPTP) [51]. Currently, melatonin shows an anti-apoptotic effect by decreasing the expression of cleaved caspase-3 and cleaved caspase-9 as well as increasing the levels of SOD and CAT in A549 cells. In addition, ERS can also be initiated by oxidative stress [52]. Therefore, melatonin alleviated the apoptosis of A549 cells induced by PM_2.5_ by inhibiting oxidative stress and ERS. Nrf2, as the central regulator of cellular oxidative stress, initiated the expression of detoxifying enzymes and antioxidant enzyme genes and increased cell resistance to electrophilic chemicals [53]. In our study, melatonin down-regulated Nrf2 expression in A549 cells exposed to PM_2.5_, which is in conflict with previous studies indicating that melatonin protected cells from oxidation by increasing Nrf2 levels [54,55]. Here, two hypotheses have been made as follows: Cancer cells, including the A549 cell, have a strong antioxidant capacity to maintain rapid division and proliferation [56]. Moreover, melatonin has been shown to have an anticancer effect in some studies [43,44,57], which may lead to a decrease in the expression of Nrf2. In addition, our results indicated melatonin enhanced antioxidant enzyme expression, and other studies also revealed that melatonin can scavenge free radicals directly [12], which contributed to maintaining cell homeostasis and further inhibiting Nrf2 expression.

**Figure 7:**
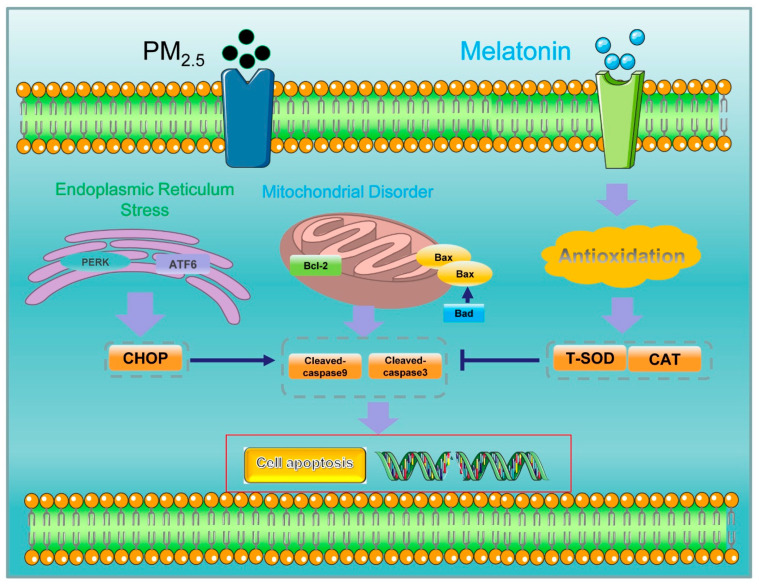
The schematic mechanism of melatonin resisting apoptosis induced by PM_2.5_ in A549 cells.

Long-term exposure to high concentrations of PM_2.5_ in poultry at different stages increased the incidence of respiratory disease, further causing poor animal growth performance and economic damage [17,18,19,20]. The wide use of antibiotics has largely raised poultry productivity and lowered poultry mortality and morbidity substantially during the food-producing process [58]. However, the application of antibiotics as feed additives in general and with other intentions not for therapy has been limited as the diverse antibiotic-resistant mechanisms of pathogenic bacteria have been generated and shared between animals and humans in conventional production, and the antibiotics were also not permitted in organic production systems [58,59]. Improving animal welfare and limiting the use of antibiotics have become priorities in animal production [60]. Spices, herbs [59], and a variety of plant extracts such as unripe raspberry extracts [61] and Annona muricata Linnaeus extracts [62] have been confirmed to have antimicrobial activity. In addition, melatonin showed immuno-enhancing activity in poultry by its immunoregulation action [63] and acted as a colistin adjuvant, avoiding mobilized colistin tolerance of Gram-negative pathogens [64], suggesting the potential alternative of melatonin to antibiotics in poultry production. The study indicated that melatonin has been used to eliminate possible physiological and metabolic abnormalities in poultry production without any side effects [65]. We demonstrate that melatonin suppressed A549 cell apoptosis by enhancing its antioxidant capacity (Figure 7) and revealed new roles in promoting poultry production. However, there are few reports focusing on clinical validation of melatonin in animal production, mainly because of the high cost of melatonin due to its complicated synthesis and extraction process [66], and melatonin use in animal husbandry production is limited to test animals. Therefore, further evaluating the physiological effect of melatonin and promoting its application during poultry production could be the focus of future research.

## 5. Conclusions

Our study indicates that melatonin alleviates A549 cell damage after exposure to chicken house PM_2.5_, which expands the potential application of melatonin in the poultry industry and promises animal welfare.

## Figures and Tables

**Table 1:** The sequences of the gene primers.

Gene	The Sequence of the Primers	Gene	The Sequence of the Primers
Bad	F: GATCGGGCTTGGGGTGAGAC R: TCATCTGTCTGCCGGGTCTG	CHOP	F: TTCACCACTCTTGACCCTGC R: TTCCTGCTTGAGCCGTTCAT
Bax	F: AGAAGCTGAGCGAGTGTCTC R: CGGAAAAAGACCTCTCGGGG	β-actin	F: GATCTTCATTGTGCTGGGTG R: GGGAAATCGTGCGTGACATT
Bcl-2	F: CTTTGAGTTCGGTGGGGTCA R: GGGCCGTACAGTTCCACAAA		
PERK	F: GCCAATGAGAGAGCAAACGC R: ATCTCGGACATCGCCCATTG		

**Table 2:** The primary components attached to PM_2.5_.

Component	Concentration (μg/m^3^)	Component	Concentration (μg/m^3^)
Organic carbon	39.65 ± 6.91	Cl^−^	4.79 ± 2.93
Elemental carbon	11.58 ± 2.50	NO_3_^-^	30.25 ± 1.33
Na^+^	0.7 ± 0.19	SO_4_^2−^	14.43 ± 2.32
NH_4_^+^	13.8 ± 0.97	Mg	0.12 ± 0.12
K^+^	1.58 ± 0.77	K	1.39 ± 0.53
Mg^2+^	0.34 ± 0.04	Ca	0.55 ± 0.29
Ca^2+^	3.23 ± 0.18	Fe	1.01 ± 0.26
F^−^	0.29 ± 0.09	Endotoxin	0.3 EU/m^3^

## Data Availability

All data in this article are presented in the article, and the original data are available from the corresponding author upon reasonable request.
